# Association between number of dissected lymph nodes and survival in patients undergoing resection for clinical stage IA pure solid lung adenocarcinoma: a retrospective analysis

**DOI:** 10.1186/s12890-023-02675-2

**Published:** 2023-10-21

**Authors:** Yu Rong, Junfeng Liu, Nianqiao Han, Zhihua Shi, Tao Jiang, Nan Zhang, Xi’e Xu, Jinhuan Yin, Hui Du

**Affiliations:** 1 Department of Thoracic Surgery, The Fourth Hospital of Hebei Medical University, 12 Jiankang Road, Shijiazhuang, Hebei 050011 China; 2 Department of Thoracic Surgery, The First Affiliated Hospital of Hebei North University, Zhangjiakou, Hebei 075000 China

**Keywords:** Lymph node dissection, Recurrence-free survival, Neoplasm staging, Adenocarcinoma of the lung

## Abstract

**Background:**

Lymph node dissection is essential for staging of pure solid lung adenocarcinoma and selection of treatment after surgical resection, particularly for stage I disease since the rate of lymph node metastasis can vary from 0 to 23.7%.

**Methods:**

We retrospectively screened all adult patients (18 years of age or older) who underwent lobectomy for pure solid cT1N0M0 lung adenocarcinoma between January 2015 and December 2017 at our center. Cox proportional hazard regression was used to assess the association between the number of dissected lymph nodes and recurrence-free survival (RFS) and to determine the optimal number of dissected lymph nodes.

**Results:**

The final analysis included 458 patients (age: 60.26 ± 8.07 years; 241 women). RFS increased linearly with an increasing number of dissected lymph nodes at a range between 0 and 9. Kaplan-Meier analysis revealed significantly longer RFS in patients with ≥ 9 vs. <9 dissected lymph nodes. In subgroup analysis, ≥ 9 dissected lymph nodes was not only associated with longer RFS in patients without lymph node metastasis (n = 332) but also in patients with metastasis (n = 126). In multivariate Cox proportional hazard regression, ≥ 9 dissected lymph nodes was independently associated with longer RFS (hazard ratio [HR], 0.43; 95% confidence interval [CI], 0.26 to 0.73; *P* = 0.002).

**Conclusions:**

≥9 Dissected lymph nodes was associated with longer RFS; accordingly, we recommend dissecting 9 lymph nodes in patients undergoing lobectomy for stage IA pure solid lung adenocarcinoma.

**Supplementary Information:**

The online version contains supplementary material available at 10.1186/s12890-023-02675-2.

## Introduction

The expansion of screening programs has allowed an increasing number of lung cancer patients to undergo surgery [1, 2]. In patients without lymph node metastasis, such as adenocarcinoma in situ (AIS) and minimally invasive adenocarcinoma (MIA), lung cancer could be practically cured [3, 4]. Once the lymph nodes are involved, however, the risk of postoperative recurrence is significantly increased [5, 6]. The standard procedure for non-small cell lung cancer (NSCLC) surgery is lobectomy plus lymph node dissection [7]. However, lymph nodal sampling is widely performed due to its convenience in the real-world settings. The non-inferiority of lymph nodal sampling compared to lymph node dissection on postoperative outcome is still controversial. Sufficient number of lymph nodes needs to be sampled to allow accurate disease staging and selection of appropriate postoperative treatment [8]. Several studies supported station-based sampling [9, 10] or lobe-specific systematic nodal dissection based on tumor histology [11], but others suggested a need for a higher and more precise number of dissected lymph nodes [12–15]. This issue is particularly important for clinical stage IA lung adenocarcinoma since the rate of nodal involvement varies substantially (0-23.7%) [16]. We conducted a retrospective analysis to examine the relationship between the number of dissected lymph nodes and recurrence-free survival (RFS) in patients with clinical stage IA pure solid lung adenocarcinoma and to clarify the number of resected lymph nodes which did not lead to worse outcome.

## Methods

### Study design and patients

**Fig. 1:**
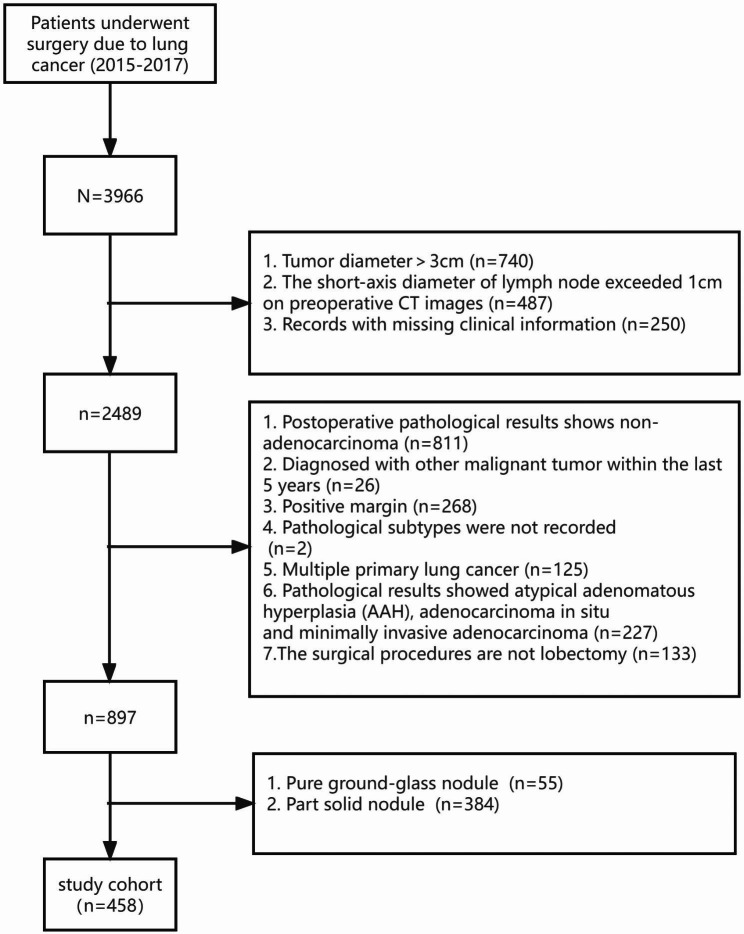
A flowchart of patient selection

We retrospectively screened all adult patients aged 18 years or older who underwent lobectomy for pure solid cT1N0M0 lung adenocarcinoma at the Fourth Hospital of Hebei Medical University during a period from January 1, 2015 to December 31, 2017. The flowchart of the subject enrollment is provided in Fig. 1. The study protocol was approved by the Institutional Ethics Review Board (2018mec160). Based on the retrospective design, informed consent was waived.

For inclusion in the final analysis, a diagnosis of primary lung adenocarcinoma must be confirmed by postoperative pathology, and preoperative computed tomography (CT) of the chest must show pure solid nodules. Patients with AIS or MIA were excluded from the analysis. Patients with part-solid nodules were excluded from the study due to the significant disparity in the rate of lymph node metastasis versus solid nodules [17].

### Preoperative staging

Clinical staging was performed according to the 8th edition of the TNM classification for lung cancer [18] and based on thin-section CT on a 128-slice spiral system (Siemens, Definition, Flash). The following parameters were used during scanning: slice thickness, 5 mm; slice interval, 5 mm; voltage, 120 kVp; scanning matrix, 512 × 512; lung window width, 1,200 HU; lung window level, -600 HU; mediastinal window width, 350 HU; mediastinal window level, 40 HU.

Two thoracic surgeons (RY and NH) independently reviewed CT scans to determine the location of pulmonary nodules, their maximum diameter and density, and the presence or absence of enlarged lymph nodes. Disagreements were resolved by a senior thoracic surgeon with 40 years of experience (JL). Pure solid nodules were defined as opacification that completely obscured the underlying vascular pattern [19]. cT1 was defined as maximum diameter at < 30 mm on chest CT. cN0 was defined as absence of non-calcified lymph nodes with a short-axis diameter of > 1 cm in the mediastinum or hilum, parabronchial or intrapulmonary parenchyma [20]. Although PET-CT can identify positive lymph nodes more accurately than CT, routine preoperative PET-CT does not seem to be widely available in various hospitals in most cities in China [21]. Therefore, we still chose CT as the screening method for enrolled patients.

### Postoperative staging and the number of dissected lymph nodes

The number of dissected lymph nodes was the sum of the mediastinal and intrapulmonary lymph nodes. Additional pulmonary lymph nodes identified during postoperative pathological examination were added to this sum at the discretion of the pathologists. The number of intact lymph nodes in the region that were clustered together after formalin fixation was calculated by the pathologist.

The pT and pN staging was defined according to the 8th edition of TNM staging system.

### Follow-up

The routine follow-up schedule at authors’ institute was every 6 months during the first 2 years after surgery and every 12 months thereafter. Each follow-up visit included a clinical examination, CT of the chest and abdomen, and a panel of serum tumor markers that consisted of carcinoembryonic antigen, squamous cell carcinoma antigen, neuron-specific enolase, carbohydrate antigen 125, and cytokeratin 19 fragment antigen 21 − 1.

If recurrence was suspected, additional examinations were performed as appropriate, such as contrast-enhanced cranial magnetic resonance imaging and radioisotope skeletal scanning. Local recurrence was defined as recurrence of cancer in the ipsilateral lung or mediastinal/hilar lymph nodes. Distant metastasis was defined as recurrence in all other part than local recurrence. RFS was calculated from the time of surgery to recurrence (either locoregional of distant) or death, whichever came first. The last follow-up was conducted in July, 2022.

### Statistical analysis

Data were analyzed using R 4.1.2 (The R Foundation, www.r-project.org) and Empower (X&Y Solutions, Boston, MA, USA). *P* < 0.05 (2-sided) was considered statistically significant. Categorical variables were analyzed by chi-squared or Fisher’s exact test and reported as number and percentage. Continuous variables following normal distribution were analyzed by Student’s t-test and reported as mean ± standard deviation. Continuous variables not following normal distribution were analyzed by Mann–Whitney U test and reported as median (interquartile range; IQR).

Cox proportional hazards regression was performed to identify independent predictors of poor RFS. Independent variables (in addition to the number of dissected lymph nodes) were selected based on previous studies and > 10% change in effect estimates [22]. The relationship between RFS and the number of dissected lymph nodes was assessed using restricted cubic spline analysis and fitted using a model based on the log-likelihood ratio. A recursive algorithm was used to identify the inflection point in the number of dissected lymph nodes. Kaplan-Meier curves of RFS were compared between the patient subgroups using the log-rank test.

Sensitivity analyses were conducted by: (1) limiting the number of dissected lymph nodes in single-station to a maximum of 5. According to a previous randomized control study, the largest numbers dissected lymph nodes in a single station were resected from stations 4R and 7, with a median of four and three nodes, all less than 5, respectively [23]. Moreover, the number of lymph nodes fragment, which could not be counted exactly, was recounted to 5. This may help minimize the interference of lymph node fragments on the count results of the number of dissected lymph nodes; (2) treating occurrence and death as competing events using the Fine-Gray subdistribution hazard regression.

## Results

### Patient characteristics

Of the 3,966 patients screened,458 (241 women) were included in the final study, with a mean age of 60.3 ± 8.1 years) (Fig. 1) (Table 1). Disease stage was IA in 238 (51.96%), IB in 94 (20.52%), IIB in 56 (12.23%), and IIIA in 70 (15.28%) patients. The most frequent histological subtype was acinar predominant, followed by solid predominant, papillary predominant, lepidic predominant and micropapillary predominant. The median number of dissected lymph nodes was 15 (range, 0–39; IQR, 11–19). Lymph node metastasis was reported in 126 (27.51%) patients. Within the median follow-up of 60.20 (IQR, 54.87–66.03) months, recurrence occurred in 118 (25.76%) patients, and 93 (20.31%) patients died.

**Table 1:** Clinicodemographic characteristics of the 458 patients in the study

Characteristic	Value
Female	241 (52.62)
Age	60.26 ± 8.07
> 65 yr	109 (23.80)
Body mass index, kg/m^2^	24.55 ± 3.08
Age-adjusted Charlson Comorbidity Index	2.00 (1.00–3.00)
Smoking status	
Never-smoker	308 (67.25)
Ex-smoker	62 (13.54)
Current smoker	88 (19.21)
Family history of malignancy	90 (19.65)
Surgical year	
2015	124 (27.07)
2016	153 (33.41)
2017	181 (39.52)
Tumor site	
RUL	143 (31.22)
RML	20 (4.37)
RLL	103 (22.49)
LUL	101 (22.05)
LLL	91 (19.87)
Histopathological subtype	
Lepidic	25 (5.46)
Papillary	52 (11.35)
Acinar	262 (57.21)
Micropapillary	4 (0.87)
Solid	74 (16.16)
Other	41 (8.95)
Number of N1 node	6.00 (4.00–8.00)
Number of N2 node	9.00 (6.00–12.00)
Number of lymph nodes examined	15.00 (11.00–19.00)
Spread through air space	115 (25.11)
pT stage	
T1a	37 (8.08)
T1b	144 (31.44)
T1c	135 (29.48)
T2	142 (31.00)
pN stage	
N0	332 (72.49)
N1	56 (12.23)
N2	70 (15.28)
pTNM stage	
IA	238 (51.96)
IA1	34 (7.42)
IA2	115 (25.11)
IA3	89 (19.43)
IB	94 (20.52)
IIB	56 (12.23)
IIIA	70 (15.28)
Postoperative adjuvant therapy	249 (54.37)
Chemotherapy	218(47.60)
Radiotherapy	1(0.22)
Targeted therapy	30(6.55)

Values are n (%), median (interquartile range) or mean ± SD, unless otherwise noted

LLL, lower lobe of left lung; LUL, upper lobe of left lung; RLL, lower lobe of right lung; RML, middle lobe of right lung; RUL, upper lobe of right lung

### Relationship between the number of dissected lymph nodes and RFS

**Fig. 2:**
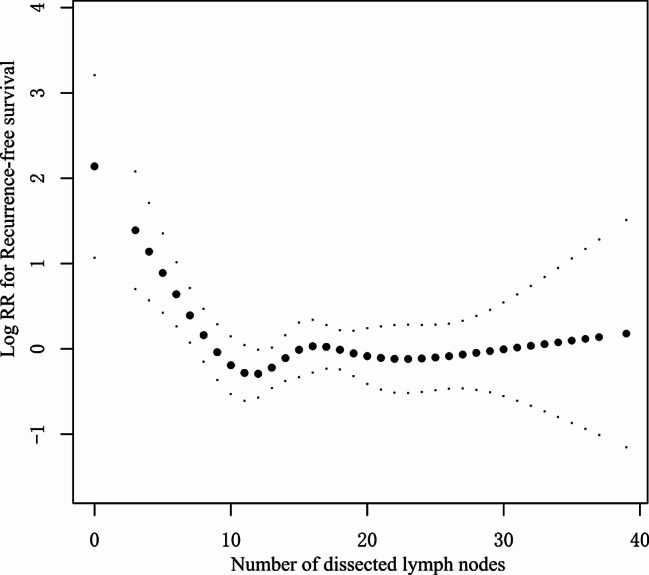
**Association between the number of lymph nodes examined and recurrence-free survival (RFS)** Adjusted for age, sex, age-adjusted Charlson Comorbidity Index, surgical year, tumor site, family history of malignancy, histopathological subtype, spread through air space, pT, pN and Postoperative adjuvant therapy

The restricted cubic spline analysis showed an L-shape relationship between the number of dissected lymph nodes and RFS (Fig. 2). The relationship was non-linear (*P* for non-linearity = 0.033). *P* for the log-likelihood ratio test for a two-line model of RFS was 0.001, indicating the presence of an inflection point. In recursive algorithm analysis, the inflection point was 9.

The following variables were included in the Cox regression as covariates: sex (male vs. female), age (≤ 65 vs. > 65 year), tumor site (RUL vs. RML/RLL/LUL/LLL), age-adjusted Charlson comorbidity index, year of surgery (2015 vs. 2016/2017), family history of malignant tumor (yes vs. no), histopathological subtype (micropapillary or solid predominant subtype vs. others), spread through air space (yes vs. no), pT stage (T1a vs. T1b/T1c/T2a), pN stage (N0 vs. N1/N2) and postoperative adjuvant therapy (yes vs. no). Within a range from 0 to 9 dissected lymph nodes, RFS increased linearly with increasing number of dissected lymph nodes (Table 2).

**Table 2:** Association between the number of dissected lymph nodes and recurrence-free survival according to piece-wise regression models

Model	Recurrence-free survival	
	HR (95%CI)	*P* value ^a^
One-line (non-segmented)	0.98 (0.95, 1.01)	0.236
Segmented regression		
Fewer than 9 nodes	0.77 (0.67, 0.87)	< 0.001
At least 9 nodes	1.01 (0.97, 1.04)	0.653
*P* in log-likelihood ratio test	0.001	

^a^ Adjusted for age, sex, age-adjusted Charlson Comorbidity Index, surgical year, tumor site, family history of malignancy, histopathological subtype, spread through air space, pT, pN and postoperative adjuvant therapy

CI, confidence interval; HR, hazard ratio

### Association of the number of dissected lymph nodes with RFS and OS

**Fig. 3:**
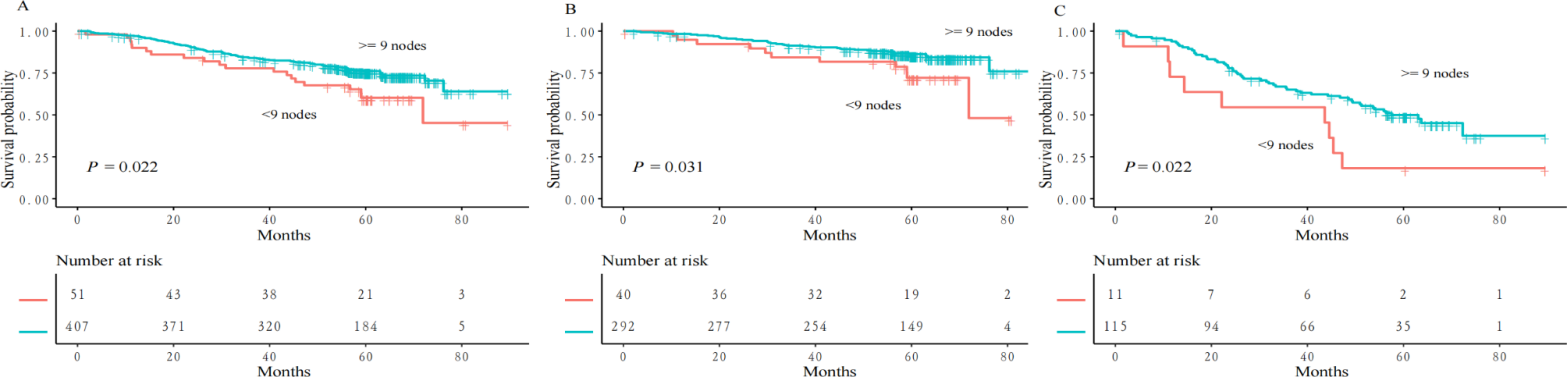
Subgroup analysis of recurrence-free survival (RFS) in (A) all patients, (B) patients without lymph node metastasis (N0) and (C) patients with lymph node metastasis (N1 or N2)

RFS was significantly longer in patients with ≥ 9 vs. <9 dissected lymph nodes (log-rank test *P* = 0.022; Fig. 3A). Such a difference was observed in the subgroup analysis that only included 332 patients without lymph node metastasis (log-rank test *P* = 0.031; Fig. 3B) as well as the analysis that only included 126 patients with lymph node metastasis (log-rank test *P* = 0.022; Fig. 3C).

In the multivariate Cox regression, the following factors were independently associated with RFS: the number of dissected lymph nodes, aCCI, family history of malignancy, pN stage, and postoperative adjuvant therapy (Table 3). The association between ≥ 9 dissected lymph nodes and improved RFS was apparent in all sensitivity analyses (Table 4).

**Table 3:** Multivariate Cox proportional hazard regression to identify independent predictors of survival

	Recurrence-free survival	
Variable	HR (95%CI)	*P* value
Number of dissected lymph nodes		
Fewer than 9	1 (reference)	
At least 9	0.43 (0.26, 0.73)	0.002
Sex		
Male	1 (reference)	
Female	1.04 (0.71, 1.53)	0.845
Age		
65 or younger	1 (reference)	
Older than 65	0.93 (0.57, 1.51)	0.775
Tumor site		
RUL	1 (reference)	
RML	1.29 (0.43, 3.89)	0.648
RLL	1.45 (0.85, 2.47)	0.170
LUL	1.51 (0.93, 2.47)	0.098
LLL	1.08 (0.61, 1.92)	0.784
Histopathological subtype		
Low-risk subtype	1 (reference)	
High-risk subtype	0.90 (0.56, 1.45)	0.666
aCCI	1.17 (1.00, 1.37)	0.047
Family history of malignancy		
No	1 (reference)	
Yes	1.66 (1.05, 2.63)	0.031
Surgical year		
2015	1 (reference)	
2016	1.56 (0.94, 2.60)	0.085
2017	1.40 (0.82, 2.40)	0.214
Spread through air space		
No	1 (reference)	
Yes	0.94 (0.60, 1.49)	0.801
pT stage		
T1a	1 (reference)	
T1b	1.57 (0.47, 5.27)	0.465
T1c	2.18 (0.66, 7.21)	0.204
T2	3.21 (0.97, 10.60)	0.055
pN stage		
N0	1 (reference)	
N1	1.47 (0.84, 2.54)	0.174
N2	3.82 (2.45, 5.96)	< 0.001
Postoperative adjuvant therapy		
No	1 (reference)	
Yes	4.15 (2.26, 7.60)	< 0.001

All variables except stratification are adjusted for in stratified results

**Table 4:** Sensitivity analyses to assess the robustness of the association between number of dissected lymph nodes and Recurrence-free survival

Number of lymph nodes examined	No. patients	No. events	Adjusted model *	
			Hazard ratio (95% confidence interval)	*P* value
Fewer than 9 ^a^	51	20	1.00 (reference)	
At least 9 ^a^	407	98	0.43 (0.26, 0.73)	0.002
Fewer than 9 ^b^	51	20	1.00 (reference)	
At least 9 ^b^	407	98	0.45 (0.27, 0.77)	0.003

^a^ Recounted the number of lymph nodes of single station which higher than 5 to 5

^b^ Death without recurrence was considered competing events for recurrence-free survival

* Adjusted for age, sex, age-adjusted Charlson Comorbidity Index, surgical year, tumor site, family history of malignancy, histopathological subtype, spread through air space, pT, pN and postoperative adjuvant therapy

## Discussion

Results from the current study suggest that 9 is the appropriate minimum number of lymph nodes that should be examined in patients with clinical stage IA pure solid lung adenocarcinoma. RFS increased in proportion to increasing number of dissected lymph nodes, but only up to 9. There was no further improvement beyond nodes. The association between improved RFS with ≥ 9 dissected lymph nodes was apparent in the subgroup analysis that not only included patients with no lymph node involvement, but also in patients with lymph node metastasis, adding support to the validity of the findings.

The issue of adequate lymph node sampling is universal for practically all cancers. For example, current guidelines recommend a minimum of 15 lymph nodes for gastric cancer [24], 8–20 lymph nodes for breast cancer depending on the T state [25], and 10–42 lymph nodes for esophageal cancer depending on the T state [26–28]. A prospective cohort study of lung cancer found that inadequate detection of intrapulmonary (N1) lymph nodes could lead to occult metastases of lymph nodes [29]. The consequence of erroneous labeling of N1 as N0 could lead to wrong decision with regards to adjuvant treatment [30, 31]. Our results showed that in patients without lymph node metastasis in postoperative pathology, the risk of recurrence decreased significantly in the group with a higher number of dissected lymph node. This may be because of the possibility that occult lymph node metastasis decreased as the number of dissected lymph node increased, and the true pathological status of the patient was more accurately assessed. Significantly, the aforementioned association remained consistent in the subgroup analysis of patients with positive lymph nodes in the current study. This finding may be attributed to the lower residual tumor burden in patient with higher number of dissected lymph nodes since more or even all metastatic lymph nodes have been resected. Patients with low number of dissected lymph nodes, in contrast, may have higher residual tumor burden after the surgery. Alternatively, detection of lymph node metastasis with a smaller number of dissected lymph node tends to suggest overall higher percentage/number of the lymph nodes with metastasis, which in turn is associated with poorer prognosis. A cohort study of 16,393 subjects with NSCLC by David and colleagues also supported the significance of higher number of lymph nodes sample in improved long-term prognosis in patients with stage I disease [32]. Unfortunately, no suggestions have been provided on the number of dissected lymph nodes in their study. A previous study of 442 patients with stage I NSCLC reported that a minimum of 6 lymph nodes needed to be examined to define lymph nodal staging and improve patient outcome [33]. The major reason for this discrepancy between this study and our findings is that their study enrolled some patients who underwent surgery before establishing rules for systematic lymph node sampling and about one-third of the enrolled patients admitted for surgery at community hospitals that lacked strict surgical quality control. The larger number in the current study (9) likely reflects the fact that the current study was conducted more recently in a more contemporary setting with improved quality control. A study based on SEER database plus a multi-center retrospective study in patients with NSCLC suggest that increasing the number of examined lymph node to 16 can result in superior overall survival [34]. Patients with stage IIIB or IV disease were excluded in their study and there was no consideration of treatment following tumor recurrence. Subsequent treatments, especially targeted therapy and immunotherapy, may have potential effects on overall survival [35, 36]. To minimize this influence, we chose recurrence-free survival as the primary outcome of our study. Different primary outcomes may be a reason for the different sampling thresholds for lymph nodes. Another reason may be the presence of T4 staging patients recruited by investigator, who may have developed more lymphatic metastasis and therefore may have contributed to the increase in numbers at the time of sampling. A study of patients with pure solid nodules with cT1a-2bN0-1M0 reported that approximately 16–17 lymph nodes were the optimal cut point for RFS [37]. This study also considered the imaging characteristics of patients when recruiting patients. However, the population enrolled in this study was NSCLC. Studies have clearly pointed to differences in the risk of recurrence between adenocarcinoma and other types of NSCLC [38]. In addition, different histologic subtypes of adenocarcinoma (especially micropapillary/solid subtypes) had a significant impact on survival outcomes [39, 40]. In patients with pure solid nodules, the high-risk subtype of adenocarcinoma has a higher chance of recurrence [41]. It is important to note the current study excluded patients with AIS and MIA, two subpopulations in which lymph node metastasis rarely occurs. Also, this study only included patients with adenocarcinoma, and the results may not apply to cases in which intraoperative pathology (wedge-resection or needle aspiration) is not performed prior to lobectomy in patients with preoperative histologic diagnosis.

The minimum number of dissected lymph nodes as derived from the current study (9) is similar to a previous study based on SEER database plus a Chinese multi-center retrospective study in patients with NSCLC [42]. The fact that we obtained the similar number despite adjusting for other variables (e.g., age-adjusted Charlson comorbidity index, year of surgery, histopathological subtype, spread through air space) adds to the robustness of the findings. Our results may be more reliable partly because we only recruited patients with cT1 (tumor diameter ≤ 3 cm), and the effect of T stage on outcomes was greatly reduced, and on the other hand, we adjusted for covariates more comprehensively. In addition, our study also incorporated the prognostic potential of imaging, which was not included in the above studies [17].

Findings from the current study also suggest that “more is better, but only to a certain extent”. Consistent with the conclusion of a large trial of the American College of Surgeons Oncology Group (ACOSOG) Z0030 [23], prognosis did not improve with higher number of dissected lymph nodes at a range beyond 9. Indeed, higher number of dissected lymph nodes has been associated with longer operation time and increased bleeding [43]. The reliability of our findings is supported by their robustness to subgroup and sensitivity analyses.

Our findings should be interpreted with caution in light of several limitations. First, the retrospective design increases the risk that our analysis is confounded by other factors for which we did not control. Second, it remains unclear whether our results can be generalized to patients whose histological subtype of adenocarcinoma cannot be determined before or during surgery. Third, this study was conducted at a single-center, and therefore must be considered as preliminary.

## Conclusion

In patients receiving lobectomy of stage IA pure solid lung adenocarcinoma, longer RFS was associated with higher number of dissected lymph nodes up to and not beyond 9 dissected lymph nodes.

### Electronic supplementary material

Below is the link to the electronic supplementary material.

Supplementary Material 1

Supplementary Material 2

## Data Availability

(ADM) All data generated or analyzed during this study are included in this published article and its supplementary information files.
