# Supplementary material for: Association between number of dissected lymph nodes and survival in patients undergoing resection for clinical stage IA pure solid lung adenocarcinoma: a retrospective analysis
Source: BMC Pulm Med. 2023 Oct 21;23:401. doi: 10.1186/s12890-023-02675-2 (PMC10590513; doi:10.1186/s12890-023-02675-2)
Supplement: Supplementary file 1 — Supplementary Material 1 [file 12890_2023_2675_MOESM1_ESM.docx]

**Supplementary Table 1.** Adjuvant therapy in patients with <9 versus ≥9 dissected lymph nodes.

|  | <9 lymph nodes | ≥9 lymph nodes | *P* value |
| --- | --- | --- | --- |
| Adjuvant therapy |  |  | 0.704 |
| No | 22 (43.1%) | 187 (45.9%) |  |
| Yes | 29 (56.9%) | 220 (54.1%) |  |
| Chemotherapy | 26 (51.0%) | 192 (47.2%) |  |
| Radiotherapy | 0 (0.0%) | 1 (0.2%) |  |
| Targeted therapy | 3 (5.9%) | 27 (6.6%) |  |

Data are presented as number (percentage).
